# Case of Uterine Hæmorrhage, Consequent upon Want of Contractile Power in the Uterus, Terminating Fatally

**Published:** 1852-08

**Authors:** A. M. Dunton

**Affiliations:** Plover, Wis.


ART. IV.—*Case of Uterine Hæmorrhage, consequent upon want of Contractile Power in the Uterus, terminating fatally.* By A. M. DUNTON, M. D., of Plover, Wis.

ON the 9th June I was called to see Mrs. W., aged 20, in her first labor. She had been in labor forty-eight hours, during most of which time her pains were reported to have been active, and her delivery was expected to be speedily accomplished. She had made great exertions to help herself, and had been encouraged from time to time till she was exhausted, and the pains had nearly ceased. Up to this time she had been attended by an old lady, who said she had done all she could and desired that a physician should be sent for.

On the evening of the second day I was sent for, and having fourteen miles to ride in a dark and rainy night did not arrive till about midnight. I found the woman complaining of much uneasiness and languor, some pain in the back, and suffering from retention of urine,—pulse 90, with thirst and pain in the head. On examination, I found the uterus dilated, the crown of the head resting on the symphysis pubes, the face to the sacrum, the membranes entire, although the woman said the water had broke the day before,—the vulva hot, dry, tender, and swollen. By raising the head with the finger she was enabled to pass a large quantity of urine, and by a little exertion in the same way, the head was reduced to nearly a natural position. After this much of the uneasiness abated, I recommended some nourishment, plenty of cool drink, the avoidance of all exertion, rest, and, if possible, sleep. During the remainder of the night she slept but little,—had no pain except some back-ache. At eight, A. M., she complained of pain in the head, which she said had been severe the day before; I then took from the arm eight ounces of blood, which relieved the head and produced more perfect intervals of rest, so that she slept occasionally; and, although she had no pain that seemed to exert any force upon the child, still in the course of the day the head descended to a level with the os externum. I now steeped one dram of pulverized ergot in a tea-cupful of hot water and gave it in three doses, at intervals of twenty minutes. This produced no effect in increasing the pains, though followed by some degree of exhilaration. After waiting an hour or more there was less complaining than before. She now urged me to give her something to make her sleep, and as there was only an indefinite uneasiness and an occasional powerless effort to bear down, I gave a dose of morphine, which produced much incoherent talk and a disposition to change her position, and, after a time, a little sleep. At three o'clock, the following morning, it became apparent that her strength would not last much longer, and as there was no prospect of pains, I proceeded to deliver. After the head was released I gave ergot ʒj in an ounce of hot water, and two spoonsful of brandy, designing to wait still longer for contractions of the uterus; but in this situation she could not be controlled, and at four o'clock she was delivered of a large child, which no doubt had been dead for some time.

She was now free from pain and uneasiness, and expressed her gratitude for what had been done. I directed the application of cold water to the abdomen, and immediately introduced my hand into the uterus, carrying a cloth saturated with spirits and cold water with it; but, notwithstanding all the means used, such as friction, and compression of the aorta, as effectually as possible, she died at sunrise without a pain. The uterus as large as before delivery and nearly filled with blood.

Thus I have given a simple history of the circumstances, treatment, and result of the case; with the hope that any of the profession who may find aught to condemn in my practice, or anything to suggest in the treatment of a similar case, will frankly communicate the same.

